# Experiences in a capital city: How we market our services

**Published:** 2012

**Authors:** Boateng Wiafe

**Affiliations:** (Community Eye Health) Regional Director for Africa, Operation Eyesight Universal

**Figure:**
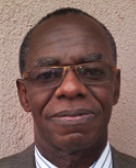
Boateng Wiafe MD MSc.

Lusaka Eye Hospital, in the capital city of Zambia, was established in 2001. We had to work hard to ensure that people knew about it and were coming forward for the eye care they needed. Here are the lessons we learnt.

- We defined our target customers. We serve mostly the west end of the city, so that is where we focus our promotion and education efforts.
- Every now and then, we are invited to speak on the radio and television about an eye condition. When we speak on the radio, we never ask patients to come to our eye hospital. The most important thing is that they seek eye care, so we advise them to go to their nearest eye clinic. Even so, many more patients usually come to us after each broadcast.
- We have a website and this also helps people to find out about our services.
- We regularly have outreach services targeted at all sections of the community. We visit institutions like the police, prisons, factories and other places of work, schools, churches, mosques, and market places. During these visits, we conduct health education and awareness creation, and screen and manage or refer those with eye conditions. We visit each community four times a year.
- Our experience shows that the majority of the patients we see were encouraged to come by people who have been to us before. This would not happen if the quality of service we offered were below standard.
- From time to time, we conduct a survey to find out about patient satisfaction. This has helped us to get better every year.
- We ask our patients if they know anyone in their neighbourhood who has an eye problem. We ask them to invite these people to come along with them to our hospital. For example, we ask patients diagnosed with glaucoma to bring their relatives for free screening.

